# ZNF667/Mipu1 Is a Novel Anti-Apoptotic Factor That Directly Regulates the Expression of the Rat Bax Gene in H9c2 Cells

**DOI:** 10.1371/journal.pone.0111653

**Published:** 2014-11-14

**Authors:** Lei Jiang, Hao Wang, Chunli Shi, Ke Liu, Meidong Liu, Nian Wang, Kangkai Wang, Huali Zhang, Guiliang Wang, Xianzhong Xiao

**Affiliations:** Department of Pathophysiology, Xiangya School of Medicine, Central South University, 110 Xiangya Road, Changsha, Hunan 410078, P. R. China; University of Cincinnati, College of Medicine, United States of America

## Abstract

ZNF667/Mipu1, a C_2_H_2_-type zinc finger transcription factor, was suggested to play an important role in oxidative stress. However, none of the target genes or potential roles of ZNF667 in cardiomyocytes have been elucidated. Here, we investigated the functional role of ZNF667 in H9c2 cell lines focusing on its molecular mechanism by which it protects the cells from apoptosis. We found that ZNF667 inhibited the expression and the promoter activity of the rat proapoptotic gene Bax gene, and at the same time prevented apoptosis of H9c2 cells, induced by H_2_O_2_ and Dox. Western immunoblotting analysis revealed that ZNF667 also inhibited Bax protein expression, accompanied by attenuation of the mitochondrial translocation of Bax protein, induced by H_2_O_2_. EMSA and target detection assay showed that the purified ZNF667 fusion proteins could interact with the Bax promoter sequence in vitro, and this interaction was dependent upon the ZNF667 DNA binding sequences or its core sequence in the promoter. Furthermore, ChIP assay demonstrated that a stimulus H_2_O_2_ could enhance the ability of ZNF667 protein binding to the promoter. Finally, a reporter gene assay showed that ZNF667 could repress the activity of the Bax gene promoter, and the repression was dependent upon its binding to the specific DNA sequence in the promoter. Our work demonstrates that ZNF667 that confers cytoprotection is a novel regulator of the rat Bax gene, mediating the inhibition of the Bax mRNA and protein expression in H9c2 cardiomyocytes in response to H_2_O_2_ treatment.

## Introduction

Zinc finger proteins are a superfamily of transcription factors. The rat zinc finger protein 667, ZNF667, provisionally named myocardial ischemic preconditioning upregulated protein 1 (Mipu1) in our lab due to its upregulation during myocardial ischemia/reperfusion, belongs to the KRAB/C_2_H_2_ zinc finger proteins that contains a KRAB domain at its N-terminus and 14 zinc fingers at its C-terminus. Both the ZNF667 mRNA and protein are expressed abundantly and predominantly in the brain and heart [1], [2]. It has also been shown that ZNF667 is a nuclear protein that is localized to the nucleus through its KRAB domain or the linker adjacent to its zinc finger region, unlike most of the KRAB/C_2_H_2_ zinc finger proteins where their zinc finger motifs are required for nuclear targeting. Like other KRAB/C_2_H_2_ zinc finger proteins, ZNF667 is a DNA binding protein and binds to the specific core sequence 5′-CTTA-3′, acting as a transcriptional repressor [3], suggesting a role in the regulation of downstream genes. Studies have shown that H_2_O_2_ induces ZNF667 expression in rat heart-derived H9c2 cells [4], [5], but to date the target gene(s) of ZNF667 is unknown. Based on informatic analysis, we have shown certain genes, including several Bcl-2 family members, contain the potential DNA sequence in their promoter regions; one of them is the Bax gene from the rat which contains up to six sites [6]. Therefore, we hypothesized that ZNF667 might be involved in Bax regulation and interfere with the apoptotic pathway in rat heart-derived H9c2 cells in response to oxidative stress.

Bcl-2 family proteins play an important role in cardiomyocyte apoptosis during oxidative stress [7]-[9]. The Bcl-2 family of proteins consists of both antiapoptotic (such as Bcl-2 and Bcl-_XL_) and proapoptotic (such as Bax and Bak) proteins [10], [11]. It has been documented that the mitochondrial apoptotic pathway is controlled by the members of Bcl-2 family [10], [11]. Bax together with Bak is a requisite gateway to the mitochondrial apoptotic machinery because cells that are doubly deficient in Bax and Bak fail to release cytochrome c and are resistant to all apoptotic stimuli that activate the intrinsic pathway [12], [13]. In healthy cells, Bax is predominantly cytosolic and binds to its antiapoptotic partners (for example Bcl-2, Bcl-_XL_), which prevents full Bax activation and apoptosis [14]. Upon cellular stress, when the capacity of its partners can be overwhelmed (for example Bax upregulation), Bax begins to oligomerize and translocates to the mitochondrial membrane. When Bax punctures the outer mitochondrial membrane (OMM), the mPTP is open and the mitochondrial contents release into cytoplasm [15]-[17]. Therefore, Bax is a major final mediator of the OMM permeabilization.

Because ZNF667 is a transcription repressor, we focused on its repressive roles in this study. Using H_2_O_2_ and Dox (doxorubicin) as stimuli, we have demonstrated that ZNF667 confers cytoprotection. We show for the first time that ZNF667 is a novel transcriptional regulator of the rat Bax gene, negatively regulating the expression of Bax by directly binding to the functional binding elements in the Bax promoter sequence in H9c2 cells.

## Materials and Methods

### Cell culture, transfection and treatment

The rat embryonic heart-derived H9c2 cells were obtained from the American Tissue Culture Collection (ATCC), and were grown in Dulbecco's modified Eagle's medium (DMEM) supplemented with 10% fetal calf serum (Gibco). Murine macrophage-like Raw264.7 cells, obtained from the Shanghai Type Culture Collection (Shanghai, China), were also grown in DMEM with 10% fetal calf serum. All cell lines were kept in an incubator at 37°C with 5% CO_2_ and 95% air in a humidified atmosphere. Transient transfection was done with Lipofectamine 2000 (Invitrogen) according to the manufacture's instructions. H_2_O_2_ was diluted in PBS, and then diluted in culture medium. Dox was directly added to the cell culture medium at the described concentrations.

### Total RNA and quantitative real-time PCR

Total RNA was extracted from H9c2 cells using the Trizol method (Invitrogen) according to manufacture's instructions. Total RNA (1µg) was reverse-transcribed using AMV Reverse Transcriptase (TaKaRa). Quantitative RT-PCR (qRT-PCR) was performed in triplicates in an ABI PRISM 7500HT System with 50 ng cDNA product, 125 nM of primers, and Fast SYBR Green Master Mix (Applied Biosystems). The primers used were listed as follows: forward 5′-CAGGAGGAATGGGAATGGC-3′ and reverse 5′- TTAGACCCTAATTCAGGACCCC-3′ for ZNF667 (194 bp), forward 5′- TGGTTGCCCTCTTCTACTTTG -3′ and reverse 5′ GTCACTGTCTGCCATGTGGG 3′ for Bax (193 bp) and forward 5′-ACTCCCATTCTTCCACCTTTG-3′ and 5′- CCCTGTTGCTGTAGCCATATT-3′ for GAPDH (105 bp). Relative expression of target genes was calculated by the 2^-△△CT^ method as previously described [6]. Final data were presented as fold changes against control.

### Construction of plasmids

**Figure 1:**
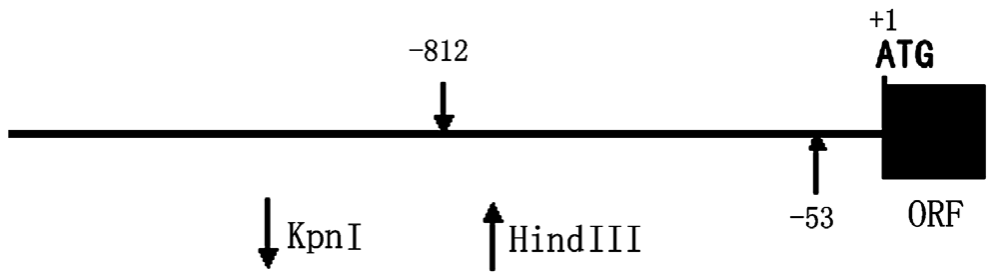
The scheme of the rat Bax gene promoter sequence. The Bax promoter sequence from -53 to -812 which contains six ZNF667 binding sites can be produced by PCR using the forward primer containing a Kpn??site and the reverse primer containing a Hind ?? site, and the PCR products can be inserted into pre-digested pGL3 basic vector to produce the luciferase reporter gene vector after digested with Kpn??/Hind ??. ATG, translation start; ORF, open reading frame.

The plasmids pcDNA3.1-ZNF667, pGEX-ZF, pEGFP-ZNF667, pEGFP-ZF, pEGFP-KRAB and pRNA-U6.1-ZNF667 (shRNA) have been constructed as previously described [3], [18]. The full-length ZNF667 (pcDNA3.1-ZNF667) DNA was used as a template and Pyrobest (Takara) was used as the DNA polymerase for the PCR amplification of the truncated ZNF667. For the construction of the reporter plasmid, the DNA sequence corresponding to the bases -812 to -53 of the rat Bax promoter (Genbank accession number: AB046392) (Fig. 1) was amplified by PCR using the following primers: 5′-GGGGTACCAAAGTAAGAGGATAAGAGAG-3′ and 5′-AAAAAGCTTTCACG TGACTCC CCGCAGAC-3′, and the PCR product was double digested with Kpn I and Hind III and inserted into pre-digested pGL3 basic vector (Promega) to produce the luciferase reporter plasmid pBa-luc, since both ends of the promoter sequence contained the sites of the two restriction endonucleases (underlined). The mutation of the putative ZNF667 binding site or its core sequence was performed by changing the core sequence CTTA to GCGC in the promoter using fusion PCR to produce the mutant luciferase reporter plasmid pBM-luc. All of the constructs were verified by DNA sequencing analysis (Invitrogen, Shanghai, China).

### Recombinant protein expression and purification

All fusion proteins were produced in Escherichia coli strain DH5α and expression was induced with 1.0 mM isopropyl β-D-thiogalactoside for 4 h according to the supplier's instructions (Amersham Pharmacia Biotech). All fusion proteins were expressed and purified as described previously [3].

### Target detection assays

Target detection assay was done essentially as described in Ref [3]. To biotin-label Bax promoter, the chemically-synthesized biotin-labeled primer pairs (See “Constructions of plasmids” described above) were used for PCR amplification of Bax promoter using the luciferase reporter plasmid pBa-luc constructed above as a template.

### EMSA

EMSA was performed as described in Ref [3]. For mutation assay, the chemically-synthesized biotin-labeled primer pairs (See “Constructions of plasmids” described above) were used for the PCR amplification of the mutant promoter sequence using the mutant luciferase reporter plasmid pBM-luc constructed above as a template to biotin-label the mutant Bax promoter sequence.

### Cell mitochondrial extraction

In order to estimate the mitochondrial Bax protein, the mitochondrial fraction was prepared using a test kit (Pierce, cat#89874) according to the manufacturer's instructions. The purity of the protein fraction was confirmed by immunoblotting with enolase (Santa Cruz Biotechnology) and VDAC (Thermo Fisher Scientific) antibodies for cytosol and for mitochondria, respectively.

### Chromatin immunoprecipitation (ChIP)

The ChIP assay was performed using a ChIP assay kit (Millipore, cat#17-295) according to the manufacturer's instructions with some modifications. Briefly, logarithmically growing H9c2 cells (1×10^8^ cells) pre-treated with 0.5 mM H_2_O_2_ for 6 h or untreated were cross-linked using formaldehyde (final concentration 1% vol/vol) in DMEM medium for 10 min on ice. After stopping the cross-linking and sonicating on ice to make soluble chromatin using an Ultrasonic homogenizer 4710(Cole-Parmer Instrument Co.), the cell lysates were diluted, precleared by incubation with protein A/G-Sepharose beads(Santa Cruz Biotechnology) and then incubated with ZNF667 polyclonal antibody we had prepared previously [4] and negative control anti-mouse-IgG antibody (Boster Biotechnologies, China) overnight at 4°C, with shaking to allow immunocomplexes to form. DNA–protein complexes were collected using protein A/G-Sepharose beads followed by several rounds of washing. Bound DNA–protein complexes were eluted from the antibodies with two incubations in elution buffer (100 mM NaHCO_3_, 1% SDS) at room temperature for 15 min. Cross-links were reversed by the addition of NaCl followed by incubation at 65°C for 4 h. RNase A and proteinase K were sequentially added and incubated for 1 h at 37°C. DNA fragments were purified using a QIAquick PCR purification kit (Qiagen, cat#28106), and used as templates for PCR amplifications. The PCR products were fractionated on 2% agarose gels, and stained with ethidium bromide. The PCR primer pairs for the ChIP were: 5′-GGGGTACCAAAGTAAGAGGATAAGAGAG-3′ and 5′-TTGCTAAGGAGTTT GAGGCAAGCC-3′, and the target sequence was 310 bp in length.

### Western blotting

After transfection, cells were cultured for 24 h. Cell lysates were collected by scraping in lysis buffer (50 mM Tris-HCl, pH 7.4, 150 mM NaCl, 1 mM EDTA, 1% Triton X-100) containing a cocktail of protease inhibitors (1× complete inhibitor). Cell debris was removed by centrifugation. Protein concentrations were determined by the Bradford assay. 20-100 µg of the protein extract was separated on 12% SDS polyacrylamide gels and electroblotted onto PVDF membranes (Millipore). The membranes were blocked with 2% bovine serum albumin (BSA) in TBS [50 mM Tris-HCl, 150 mM NaCl, pH 7.5] at 4°C for 16 h or overnight. The blocked membranes were incubated with primary antibodies in 2% BSA in TTBS [0.05% Tween 20 in TBS] for 1 to 4 h at room temperature. ZNF667, Myc tag, Bax, VDAC and β-actin were detected using rabbit polyclonal ZNF667 antibody generated in our previous study [4], rabbit monoclonal antibody against Myc tag (Cell signaling technology), rabbit polyclonal antibody against human Bax (BD Pharminogen), rabbit polyclonal antibody against VDAC (voltage-dependent anion channel protein) (Thermo Fisher Scientific) and rabbit monoclonal antibody against human β-actin (Cell signaling technology), respectively. The secondary antibodies were donkey anti-rabbit Ig-HRP (Boster Biotechnologies). Secondary antibodies were prepared in 5% milk TTBS and incubated with blots for 1 h at room temperature. The detection was performed using ECL-plus detection reagent according to the manufacturer's instructions (Amersham) or with DAB (diaminobenzidine) staining kit (Boster Biotechnologies, cat#AR1021) according to the manufacturer's instructions.

### Apoptosis detection

H9c2 cells were transfected and treated as described. For flow cytometry analysis, cells were harvested by trypsinization, washed twice with ice-cold PBS, resuspended in ice-cold PBS, and fixed with 70% ethanol. Then the cells were incubated with 1 mL of PI/Triton X-100 staining solution (0.1% Triton X-100 in PBS, 0.2 mg/mL RNase A, and 10 µg/mL propidium iodide) for 30 min at room temperature. The stained cells were analyzed using a FACScan flow cytometry in combination with BD Lysis II Software (Becton Dickinson). Ten thousand events were analyzed for each sample. To analyze morphological changes in nuclei, the cells were fixed in 4% paraformaldehyde-phosphate-buffered saline, stained with Hoechst 33258 (10 µg/ml) for 10 min, and washed three times with phosphate-buffered saline. The stained nuclei were visualized under a fluorescent inverted microscope. Caspase-3 activity was measured using a kit (Beyotime, cat#C1115) according to the manufacturer's instructions.

### Reporter gene assay

For reporter gene analysis, 1.5×10^5^ Raw264.7 cells were plated on 24-well plates and 0.5µg of pBa-luc or pBM-luc, 0.1-1µg of ZNF667 (or its truncated plasmids) and 20 ng of PRL-TK control plasmid (Promega) were co-transfected into cells by Lipofectamine 2000 reagent (Invitrogen) according to manufacturer's directions. Cells were collected 24 h after transfection, and firefly and Renilla luciferase activities were measured consecutively with a Dual-Luciferase Reporter assay reagents kit (Promega, cat#E1910) according to manufacture's recommondations using Lumat LB 9507 (Berthold Technologies). Firefly luciferase activity was normalized to Renilla luciferase activity. All assays shown were repeated at least four times.

### Statistical analysis

Data are expressed as the means ± SEM. Each experiment was performed at least three times, and the difference among three or more groups was analyzed with ANOVA and Student–Newman–Keuls post hoc test. P<0.05 was considered significant.

## Results

### ZNF667 negatively regulates the expression of the Bax gene

**Figure 2:**
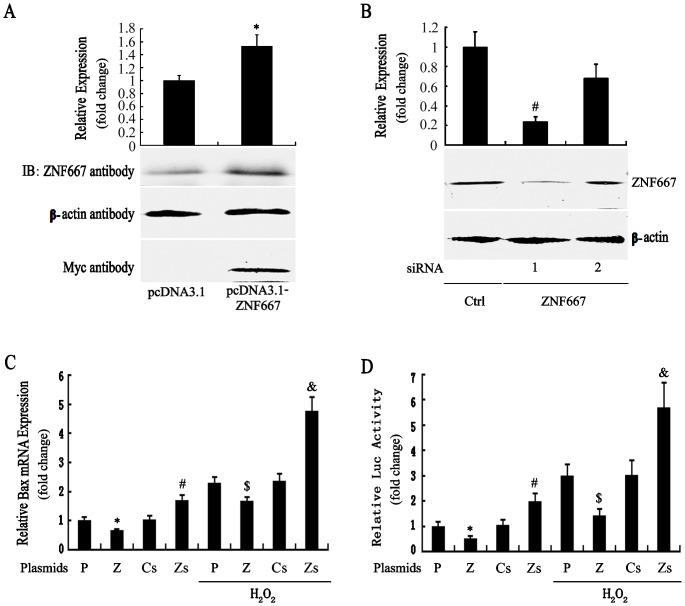
ZNF667 represses the Bax gene expression in H9c2 cardiomyocytes. Cells were transfected with plasmid pcDNA3.1 or pcDNA3.1-ZNF667, and transfected with pRNA-U6.1 or pRNA-U6.1-ZNF667 (shRNA), as indicated. Immunoblotting showing the effect of ZNF667 gene transfection (**A**) or ZNF667 siRNA (**B**) on expression of ZNF667 protein using the anti-ZNF667 and/or anti-myc tag antibodies (n = 3). Upper panel showing densitometry analysis of ZNF667 band against β-actin band. *P<0.05 vs. pcDNA3.1, #P<0.05 vs. control siRNA. After transfected with the indicated vector, or co-transfected with the indicated vector and the reporter construct pBa-luc plus pRL-TK, and serum starved in DMEM overnight, the cells were treated with 0.5 mM H_2_O_2_ for 12 h or untreated. qRT-PCR was performed to quantify Bax mRNA expression levels, and shown as the relative difference from the pcDNA3.1 or control siRNA normalized to GAPDH expression levels (n = 4, triplicate for each sample) (**C**). The reporter activity is shown as the relative luciferase activity normalized to the pRL-TK activity (n = 8) (**D**). *P<0.05 vs. pcDNA3.1; #P<0.05 vs. ctrl siRNA; $P<0.05 vs. pcDNA3.1+ H_2_O_2_; &P<0.05 vs. ctrl siRNA + H_2_O_2_. IB, immunoblot; P, pcDNA3.1; Z, pcDNA3.1-ZNF667; Cs, control siRNA; Zs, ZNF667 siRNA; Luc, luciferase.

Our previous studies have reported that H_2_O_2_ could increase the Bax expression [17], [19], [20]. In this study, we performed qRT-PCR with ZNF667-overexpressed or –knockdowned cells to determine if ZNF667 has any effect on the Bax gene expression. As shown in Fig. 2A and B, there was a significant increase or a significant reduction in ZNF667 protein levels after ZNF667 gene transfection or transfection with ZNF667 siRNA (#1) compared to the respective control. In cells transfected with ZNF667 plasmid (pcDNA3.1-ZNF667) or ZNF667 siRNA (pRNA-U6.1-ZNF667), there was a significant decrease or increase in Bax mRNA levels compared to control plasmid (pcDNA3.1) (Fig. 2C, bar 1 vs.2) or control siRNA (pRNA-U6.1) cells (Fig. 2C, bar 3 vs.4), respectively, which turns out contrary to ZNF667 levels. H_2_O_2_ largely induced Bax mRNA expression (Fig. 2C, bar1 vs. 5). However, the expression of Bax in H_2_O_2_-treated cells transfected with pcDNA3.1-ZNF667 was lower than that in the treated cells transfected with pcDNA3.1 (Fig. 2C, bar 5 vs.6). In contrast, Bax in the H_2_O_2_-treated cells transfected with ZNF667 siRNA (#1) was further induced to near 200% of that in the treated cells transfected with control siRNA (Fig. 2C, bar 7 vs.8). These results indicate that ZNF667 could inhibit the Bax gene expression induced with or without H_2_O_2_ in H9c2 cells.

To further determine the possibility that ZNF667 regulates Bax transcription, we cloned the rat Bax gene promoter (Genbank accession number: AB046392, -812 to -53) and performed a reporter gene assay. As shown in Fig. 2D, the Bax promoter activity was inhibited by ZNF667 overexpression, but it was promoted by ZNF667 knockdown. The Bax promoter activity was induced by H_2_O_2_ treatment, but the induction was inhibited by ZNF667 overexpression, and promoted by ZNF667 knockdown (Fig. 2D). These data are similar to those of Bax mRNA levels as described above. Taken together, all these results suggest that ZNF667 could inhibit Bax transcription perhaps via repressing its promoter activity.

Besides, we performed experiments with Dox-treated H9c2 cells. After 1 µM Dox was used to treat H9c2 cells, Bax mRNA levels were induced, accompanied with an increase in Bax promoter activity, but pre-transfection of pcDNA3.1-ZNF667 inhibited this induced expression and the activation of the promoter (Fig. S1). In contrast, ZNF667 siRNA increased Bax expression and its promoter activation, induced or non-induced (Fig. S1), which is similar to those of H_2_O_2_ treatment.

### ZNF667 inhibits Bax protein expression and mitochondrial translocation induced by H_2_O_2_

**Figure 3:**
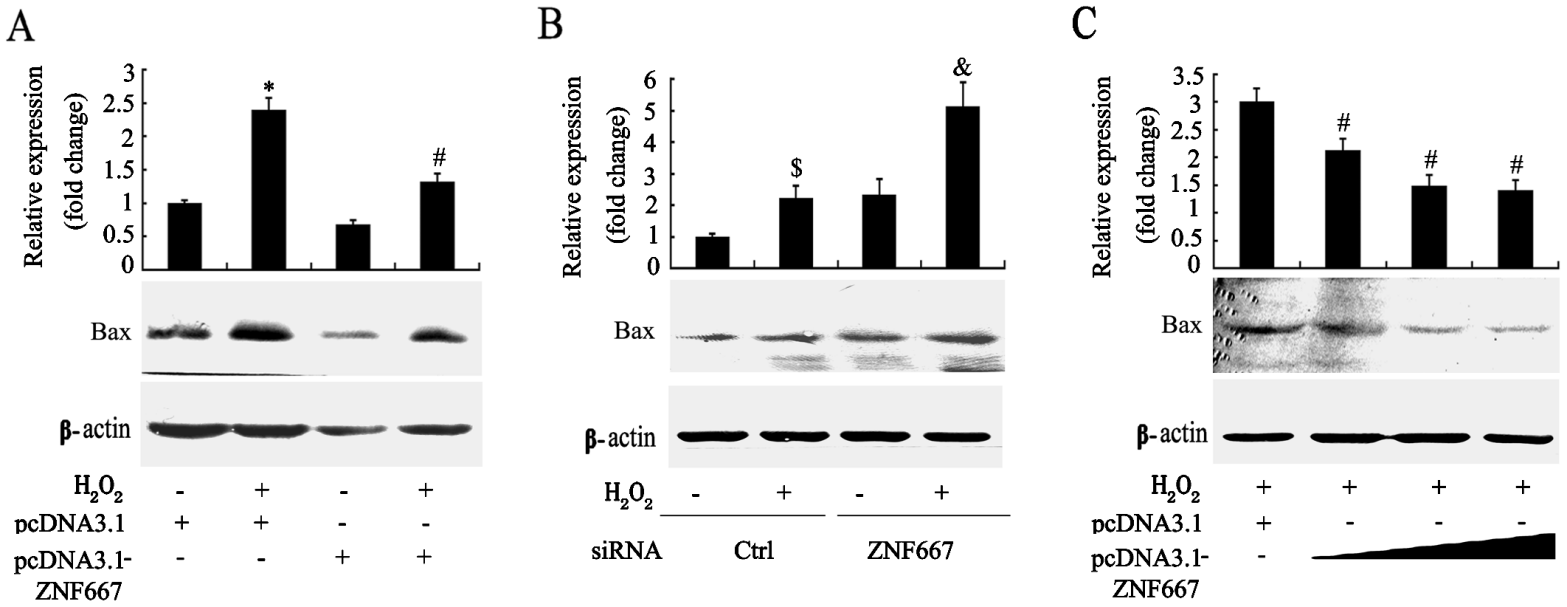
ZNF667 inhibits Bax protein expression in H9c2 cells. After cells were transfected with pcDNA3.1 or pcDNA3.1-ZNF667 (**A**), transfected with pRNA-U6.1 or pRNA-U6.1-ZNF667 (shRNA) (**B**), and transfected with different doses of pcDNA3.1-ZNF667 plasmid (4-24 µg) (**C**), respectively, for 24 h, the cells were treated with or without 0.5 mM H_2_O_2_ for another 24 h. Total proteins were extracted and subjected to SDS-PAGE followed by Western blotting analysis (n = 3). For A, B and C, shown in bottom panel are representative results, and the semi-quantitative analysis of Bax band against β-actin shown in top panel. *P<0.05 vs. pcDNA3.1; #P<0.05 vs. pcDNA3.1+ H_2_O_2_. $P<0.05 vs. ctrl siRNA; &P<0.05 vs. ctrl siRNA + H_2_O_2_.

The above results support the hypothesis that ZNF667 may directly inhibit the expression of the Bax gene. To observe the effect of ZNF667 on Bax protein expression, Bax protein was detected by Western blotting assay. In cells transfected with pcDNA3.1-ZNF667, Bax protein was reduced compared to control plasmid (pcDNA3.1) cells (Fig. 3A, lane1 vs. 3, p<0.05) as its mRNA did. H_2_O_2_ significantly induced the expression of Bax protein (Fig. 3A, lane1 vs. 2). However, ZNF667 overexpression in part attenuated the inducible expression compared with the control (Fig. 3A, lane 2 vs 4). By contrast, Bax protein levels were largely upregulated in the cells transfected with ZNF667 siRNA (#1) (Fig. 3B, bar 1 vs.3) or in the cells transfected with ZNF667 siRNA (#1) plus H_2_O_2_ (Fig. 3B, bar 1 vs. 4) compared to the control siRNA, but Bax protein expression in cells treated with the combination of ZNF667 siRNA and H_2_O_2_ was much more than that treated with the control siRNA plus H_2_O_2_ or ZNF667 siRNA alone (Fig. 3B, bar 4 vs. 2 or 3). These results indicate that ZNF667 inhibits Bax protein expression induced or non-induced by H_2_O_2_.

To further observe the effect of ZNF667 on Bax protein expression, differing amounts of ZNF667 expression plasmid were used to transfect cells. As shown in Fig. 3C, Bax protein levels were decreasing with the increasing plasmid concentrations in the range used here. These results suggest that ZNF667 can inhibit the expression of Bax protein in a dose-dependent manner in the H_2_O_2_-treated H9c2 cells.

The experiments were also performed with Dox-treated cells. ZNF667 overexpression inhibited in part Bax expression, and ZNF667 siRNA increased the Bax protein levels (data not shown), similar to the results mentioned above in H_2_O_2_-treated cells.

Other researchers reported that H_2_O_2_/Dox could mediate the translocation of Bax protein to mitochondria [21], [22]. To investigate if ZNF667 affects the mitochondrial translocation of Bax protein, a cytosol-free mitochondrial fraction was prepared. As shown in Fig. S2, after H9c2 cells were treated with H_2_O_2_, there was a marked increase in the mitochondrial Bax protein. However, the increase was significantly attenuated by ZNF667 upregulation, and promoted by ZNF667 downregulation. In healthy cells, predominantly as a cytosolic monomer, Bax protein binds to prosurvival relatives such as Bcl-2, which blocks Bax activation and its mitochondrial translocation [13]. Upon oxidative stress, Bax expression increases, which may overwhelm the capacity of its antiapoptotic partners to bind it, leading to its comformational change and mitochondrial translocation [9], [10], [13]. Thus, ZNF667 inhibits the mitochondrial translocation of Bax protein induced by H_2_O_2_ at least in part through suppressing the expression of Bax protein in H9c2 cardiomyocytes.

### Recombinant ZNF667 protein can interact with the Bax promoter in vitro

**Figure 4:**
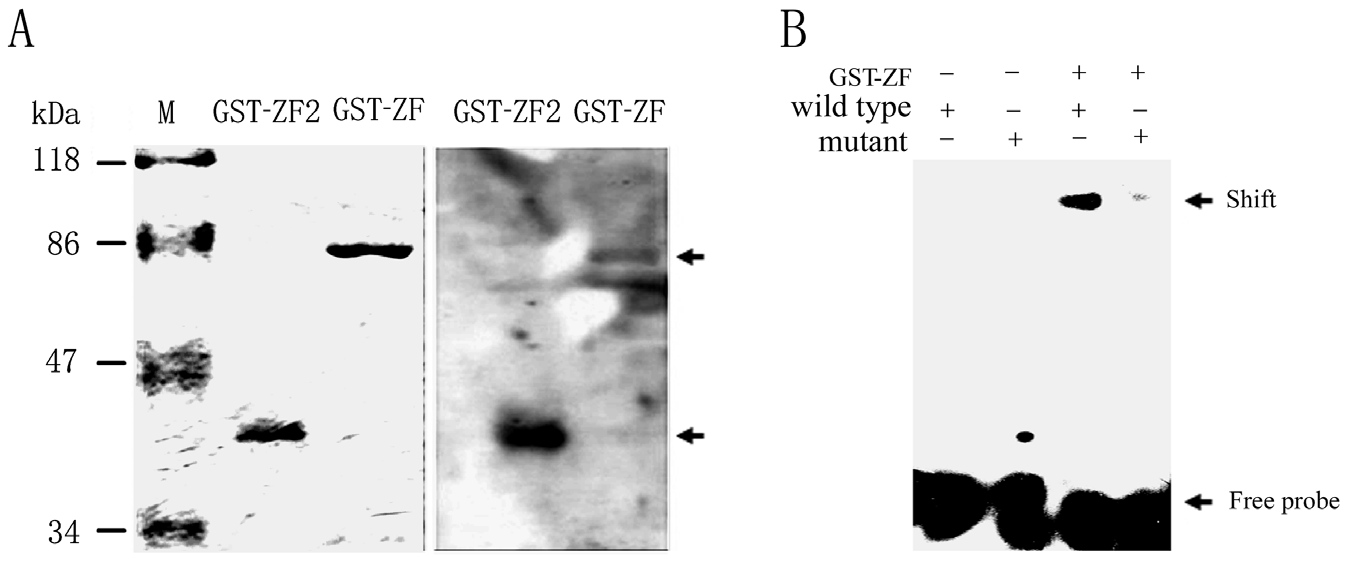
Purified GST-ZNF667 fusion proteins interact with the Bax gene promoter in vitro. (A) Purified GST-ZF, GST-ZF2 were separated on 10% SDS-PAGE and stained with Coomasie blue (left panel). A target detection assay was performed after the same proteins in A were transfected onto a nitrocellulose membrane. Biotin-labeled DNAs were hybridized to ZF and ZF2 of ZNF667. Arrows represent ZF-DNA complex (upper) and ZF2-DNA complex (lower), respectively (right panel). (B) Purified GST-ZF was added into the binding buffer containing the Bax promoter sequence or the mutant Bax sequence. After incubated at 30°C for 30 min, the mixtures were loaded onto a non-denatured electrophoresis. For both A and B, the biotin-labeled DNA-protein complexes were detected by a lightshift chemiluminescent EMSA kit. EMSA, electrophoretic mobility shift assay.

Being a transcriptional repressor, ZNF667 could inhibit the expression of the rat Bax gene and protein, which enticed us to investigate whether the Bax gene is one of its downstream genes and regulated directly. Based on bioinformatic analysis, we found that the promoter sequence of the Bax pro-apoptotic gene contains up to six putative ZNF667 binding sites or its core sequence CTTA, and the transcription factor ZNF667 regulates the expression of the Bax gene perhaps by directly binding to the promoter. In order to test this possibility, we first investigated if the ZNF667 protein can bind to the Bax promoter. We expressed and purified ZNF667 truncated fusion proteins. Using the purified ZF and ZF2 fusion proteins, the target detection assay was performed to test whether or not they could bind the Bax promoter. After the membrane was visualized with Pierce Lightshift chemiluminescent EMSA kit (Pierce, cat#20148), we were able to compare the membrane and the SDS-PAGE gel stained with Coomassie blue. As shown in Fig. 4A, there was a single distinct binding band present in either the lane containing the ZF fusion protein corresponding to 80 kDa or the lane containing the ZF2 fusion protein corresponding to 46 kDa, suggesting that both ZF and ZF2 fusions could form a protein-DNA complex with the Bax promoter in vitro, indicating that ZNF667 could bind to the promoter perhaps through its binding sequence.

To further investigate if formation of the protein-DNA complex is dependent on the core sequence, a mutated Bax promoter sequence in which CTTA was substituted with GCGC was used for the binding analysis by EMSA. As shown in Fig. 4B, although the wild type promoter sequence could interact with the ZF fusion protein, displaying a distinct band, the mutant promoter sequence and the ZF fusion could not form a protein-DNA complex, and no band was observed (Fig. 4B, lanes 3 vs. 4), indicating the binding of ZNF667 protein and the Bax promoter via the core sequences.

The results showed that ZF region of ZNF667 is sufficient for DNA binding (Fig. 4), consistent with our previous report [3]. The ZF was fractioned into two parts, namely, ZF1 and ZF2. Similarly, using the target detection assay with the purified ZF truncated fusions (ZF1 and ZF2) and the DNA probe, we demonstrated that the last six ZFs (ZF2) of ZNF667 protein at its C-terminus is necessary and sufficient for its DNA binding while the other eight ZFs (ZF1) is not required for the binding (Fig. S3).

### ZNF667 protein interacts with the Bax promoter within cells and the interaction is enhanced by H_2_O_2_

**Figure 5:**
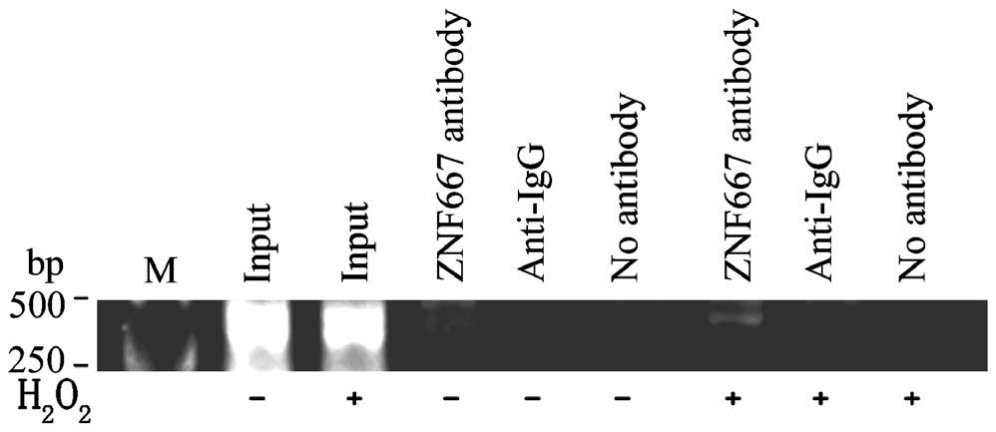
ZNF667 protein binds to the Bax gene promoter within H9c2 cells. ChIP assays were performed with H9c2 cells with indicated antibodies or without antibody as a negative control. The precipitated DNA was amplified by PCR, and the PCR products were separated on agarose gels, stained with ethidium bromide and visualized under an ultraviolet light using a gel imaging system. Input lanes show products after PCR amplification of chromatin DNA prior to immunoprecipitation. Anti-IgG, secondary antibody.

Protein-DNA binding in vitro does not necessarily mean binding within cells. In order to investigate the probability of ZNF667 binding to the Bax promoter within cells, ChIP was performed using the anti-ZNF667 antibody to pull down all the DNA that physically interacted with the ZNF667 protein, from H_2_O_2_-treated or untreated H9c2 cells. DNA was extracted from the precipitated complex, and PCR was performed to detect the presence of the Bax promoter sequence from -53 to -812, which contains six binding sites. The result showed that, in the H_2_O_2_-treated H9c2 cells, the Bax promoter was found in the immune-complex pulled down by the anti-ZNF667 antibody, but not present in the precipitation that was pulled down by the control IgG or when no antibody was added (Fig. 5, right part), indicating that ZNF667 is interacting with the Bax promoter. Similarly, Bax was not observed in all the other precipitated complexes from the H_2_O_2_-untreated H9c2 cells besides the complex pulled down by the anti-ZNF667 antibody (Fig. 5, left part). However, there was an increase in ZNF667 bound to the Bax promoter in response to H_2_O_2_ treatment compared to untreated cells (Fig. 5, lanes 4 vs.7). Therefore, the above experiments demonstrated that, although ZNF667 binds to Bax promoter sequence, a stimulus, such as H_2_O_2_ enhanced its binding within cells.

### ZNF667 directly represses the rat Bax promoter activity

**Figure 6:**
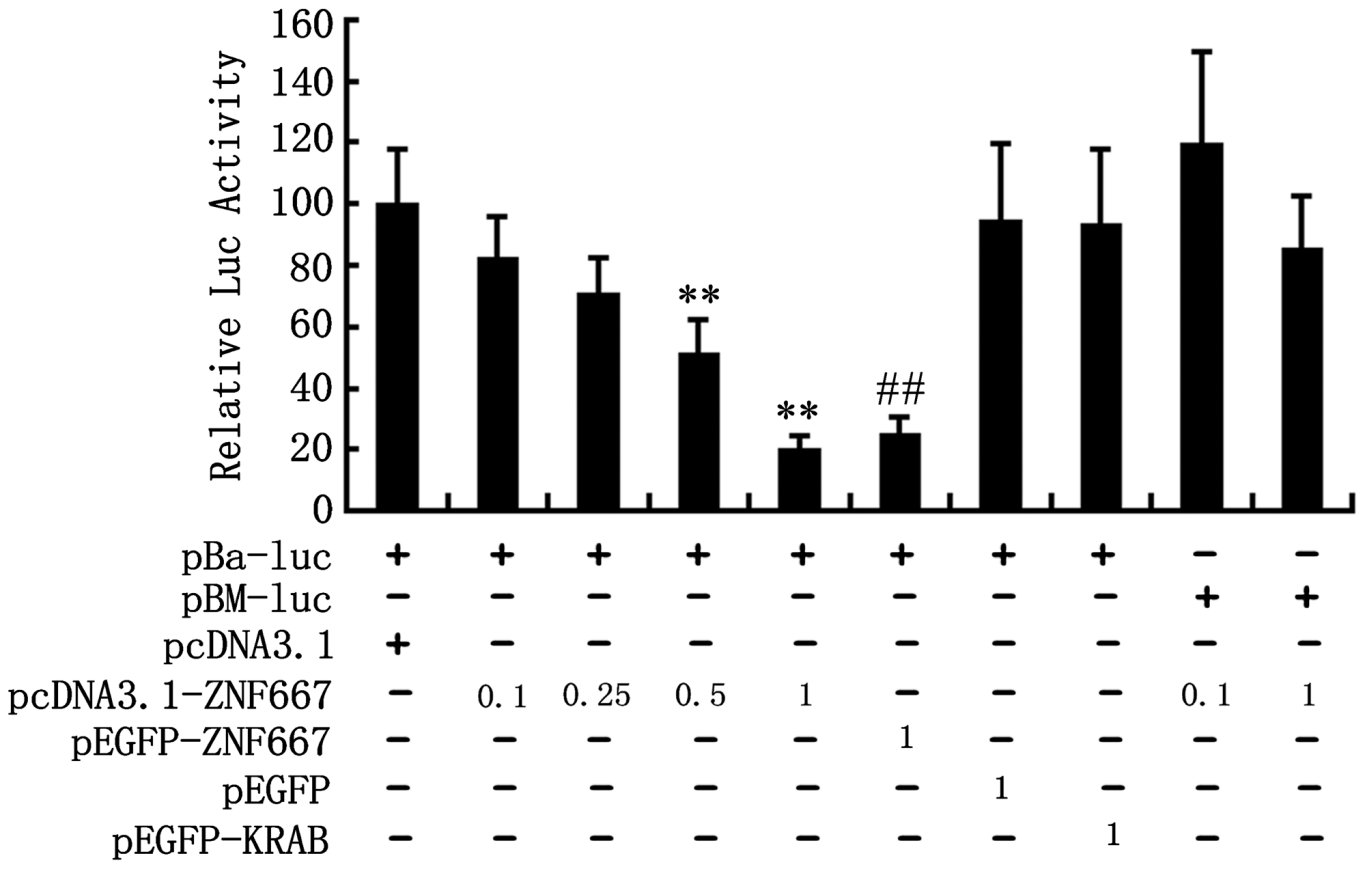
ZNF667 represses the activity of firefly luciferase driven by the Bax promoter. Bax promoter-driven reporter gene (pBa-luc) was co-transfected into Raw264.7 cells with increasing amounts (0.1-1µg ) of pcDNA3.1-ZNF667, or 1µg of ZNF667-pEGFP or truncated ZNF667(ZF-pEGFP and KRAB-pEGFP), or pcDNA3.1 empty vector. Site-mutated reporter gene (pBM-luc) was also co-transfected into Raw264.7 cells with 0.1 or 1µg of pcDNA3.1-ZNF667 plasmid. Resultant luciferase activities are expressed as relative luciferase activities normalized to the pRL-TK activity. **P<0.01 vs. pcDNA3.1; ##P<0.01 vs. pEGFP. Luc, luciferase.

In order to quantitatively analyze the effect of ZNF667 overexpression on the Bax promoter, we constructed a luciferase reporter gene vector (pBa-luc) by inserting the Bax promoter into the pGL3 basic vector, in which the Bax promoter was located upstream of the firefly luciferase gene driving expression of firefly luciferase. We then constructed a mutation vector (pBM-luc), in which all the core sequences were mutated by nucleotide substitution from 5′-CTTA-3′ to 5′-GCGC-3′, as reported previously [3]. We performed studies to determine whether ZNF667 was transcriptionally repressive in a DNA binding-dependent manner. We examined the effects of ZNF667 expression on the activity of the Bax promoter containing either the ZNF667 DNA or the mutant DNA binding sequence. The construction scheme of the Bax promoter luciferase reporter vector used in these assays is shown in Fig. 1. As seen in Fig. 6, the co-transfection of RAW264.7 cells with a ZNF667 expressing plasmid (pcDNA3.1-ZNF667 or pEGFP-ZNF667) and the Bax promoter construct, which contained six ZNF667 core sequences (pBa-luc) could repress the promoter activity in a dose-dependent manner (Fig. 6, bars 2-5), whereas the co-transfection of the cells with pcDNA3.1 empty vector and the same reporter could not repress the promoter activity (Fig. 6, bar 1 vs. 4). Co-transfection of RAW264.7 cells with either pEGFP or pEGFP-KRAB and the same reporter construct failed to reduce the activity from the reporter gene promoter construct (Fig. 6, bars 7 and 8 vs. 6), suggesting that ZNF667 inhibited the activity of the firefly luciferase gene by inhibiting the Bax promoter, and this inhibition requires its intact structure. However, co-transfection of RAW264.7 cells with pcDNA3.1-ZNF667 and an all-binding-site-mutant ZNF667 binding sequence-reporter construct (pBM-luc) also failed to reduce the activity from the reporter gene promoter construct in both high and low doses (Fig. 6, bar 9 vs.2 and bar 10 vs.5), suggesting that ZNF667 inhibited the activity of the firefly luciferase gene by direct binding to the Bax promoter through its binding sequence. These results suggest that ZNF667 has transcription repressor activity on the Bax promoter, and this repression is dependent upon its binding to the promoter via the specific DNA sequence.

### ZNF667 prevents H9c2 cells from H_2_O_2_/Dox-induced apoptosis

**Figure 7:**
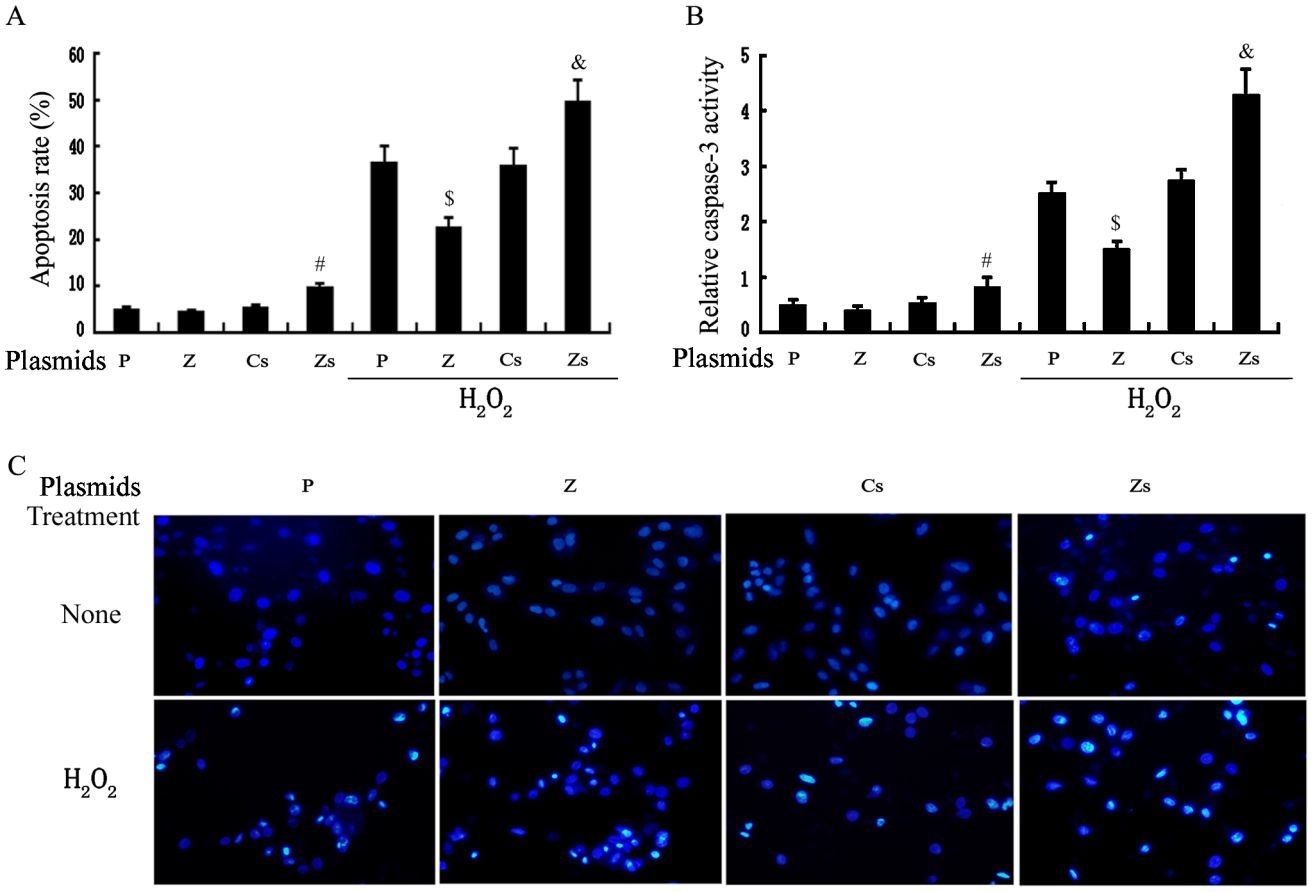
ZNF667 inhibits H_2_O_2_-mediated apoptosis and caspase-3 activation in H9c2 cells. Cells were transfected with the indicated plasmids for 24 h and followed by treatment with or without H_2_O_2_ for 6 h as indicated. The cells were used for apoptosis analysis by flow cytometry (A) or for caspase-3 activity analysis by a kit (B). #P<0.05 vs. siRNA Ctrl; $P<0.05 vs. pcDNA3.1+ H_2_O_2_; &P<0.05 vs. Ctrl siRNA + H_2_O_2_. (C) Treated as described above, the cells were stained with Hoechst 33258, and observed by fluorescence microscopy. Original magnifications: ×200. P, pcDNA3.1; Z, pcDNA3.1-ZNF667; Cs, control siRNA; Zs, ZNF667 siRNA.

It has been demonstrated that H_2_O_2_-induced apoptosis in cardiomyocytes is involved in the mitochondrial intrinsic pathway, in which Bax is an important pro-apoptotic gene associated with the mitochondrial apoptotic signal pathway. In our above results, we have demonstrated that ZNF667 could inhibit the expression of Bax at both the mRNA and protein levels, blocking Bax translocation from cytoplasm to mitochondria, indicating that ZNF667 may confer cytoprotection against oxidative stress-mediated apoptosis. To determine this possibility, we detected cell apoptosis using flow cytometry, Hoechst staining and caspase-3 activity measurement by a kit. As shown in Fig. 7 A, B and C, H_2_O_2_ treatment led to a significant increase in the apoptosis rates, a paralleled increase in caspase-3 activities and more apoptotic nuclei (the condensed, fragmented and degraded nuclei), but upregulation of ZNF667 attenuated in part the apoptosis induction and the apoptotic enzyme activation. However, downregulation of ZNF667 increased the apoptosis rates and the enzyme activity, reversing the effects of upregulation.

In addition, we also tested the possibility that ZNF667 inhibits Dox-mediated apoptosis because it inhibits Bax expression induced by Dox. As expected, ZNF667 upregulation/downregulation inhibited/promoted Dox-mediated apoptosis and caspase-3 activation in H9c2 cells (Fig. S4), which is similar to those from H_2_O_2_-treated H9c2 cells. Taken together, all these results suggest that ZNF667 could protect H9c2 cells from H_2_O_2_/Dox-induced apoptosis, and the molecular mechanism may be involved in its inhibiting Bax expression.

## Discussion

During myocardial ischemia or reperfusion, the expression of many genes such as c-fos, c-jun, junB, Egr-1, and HSP70 is upregulated [23], [24], and some of them are considered to be involved in the endogenous cardioprotection against myocardial ischemia/reperfusion injury. ZNF667 gene, then called Mipu1 (GenBank Accession No. AY221750), was isolated and cloned by Yuan and colleagues at our lab as a novel gene, which was characterized by a KRAB domain at the N-terminus and 14 successive C_2_H_2_-type zinc-finger domains at the C-terminus and was up-regulated in rat heart after a transient I/R procedure [25]. Further important observations support a central role for ZNF667/Mipu1 in the survival of C2C12 myogenic cells, because ZNF667 overexpression could reduce the growth arrest induced by serum withdrawal [26]. It was also shown that ZNF667 protein was localized to the nucleus of H9c2 cardiomyocytes and was upregulated after the cells were treated with H_2_O_2_ [2]-[5]. These observations indicated that ZNF667 might play a role in maintaining cell homeostasis and protecting the cells from being injured by oxidative stress. Previously, we have described that ZNF667 acts as a transcriptional repressor [3], even using the GAL4 system [18]. However, its downstream gene(s) which is regulated directly and could help us to explain its physiological/pathophysiological functions is unknown, and little is known about whether ZNF667 plays a role in Dox-treated H9c2 cardiomyocytes.

Because ZNF667 is a transcription repressor, we focused on its transcriptional repression in this study. Because ZNF667 is an antiapoptotic factor and because Bax is an important pro-apoptotic gene associated with the mitochondrial apoptotic signal pathway the promoter of which contains ZNF667 binding sites, Bax became our first target. In our sequence-based analysis of the rat Bax gene promoter, there was six potential ZNF667 binding sites located on the promoter sequence ranging from -813 to -52 (Fig. 1). In order to investigate the relationship between ZNF667 and Bax, we cloned the Bax gene promoter and, using the H9c2 cardiomyocytes or Raw 264.7 cells, we identified the repressive effect of ZNF667 on the promoter. The Bax expression and its promoter activity were inhibited by ZNF667 overexpression but promoted by ZNF667 knockdown (Fig. 2 and 3). Using the purified ZNF667 fusion proteins (GST-ZF and GST-ZF2), we showed that the ZNF667 could interact with the Bax promoter in vitro (Fig. 4), and that nucleotide substitutions in the ZNF667 binding sequences could abolish the in vitro protein-DNA interaction (Fig. 4B), indicating the specific interaction between the ZNF667 protein and the Bax promoter. H_2_O_2_ could enhance the affinity between the ZNF667 protein and the Bax promoter within the cells (Fig. 5). Moreover, nucleotide substitutions in ZNF667 binding sites of the Bax promoter directly affected the inhibitory effect of ZNF667 on the promoter activity (Fig. 6). To our knowledge, this study is the first to identify a specific downstream target gene of this novel KRAB/C_2_H_2_ zinc finger protein.

To observe which of the fourteen zinc fingers of ZNF667 are required for its DNA binding, we also expressed and purified the ZF1 fusion containing the eight ZFs adjacent to the N-terminus and the ZF2 fusion containing the six ZFs at the C-terminus (Fig. S3A). Using the two fusion proteins, we performed the target detection assay. The results showed that ZF2 is required and sufficient for ZNF667 binding to the DNA sequence (Fig. S3). Generally, only three, or even two zinc fingers, are sufficient for binding to the DNA sequence [27], [28]. However, ZNF667 is localized in the nucleus not by its zinc fingers but by its KRAB domain, unlike other KZNFs [3]. Therefore, more than eight zinc fingers can be used for protein-protein interactions and/or RNA-protein interactions [29]-[33]. If this is true, ZNF667 may play many other roles by its interactions with other molecules in addition to its transcriptional functions. Like other KZNFs [33], [34], ZNF667 plays a repressive role by recruiting the co-repressor KAP-1 through its KRAB domain (data not shown).

In the present study, Dox/H_2_O_2_ was used as our research tools because ZNF667 responds to H_2_O_2_ treatment as reported in our previous study and because Dox is an inducer of cardiomyocyte apoptosis. Bax is a proapoptotic factor and overexpression of Bax increases apoptosis in cardiomyocytes and other cells [35], [36]. Since ZNF667 directly contacts the Bax promoter, repressing the promoter activity and inhibiting Bax expression during Dox/H_2_O_2_ treatment, it was inferred that ZNF667 should block H_2_O_2_-induced translocation of Bax protein to mitochondria, which was then demonstrated by the experiments. Because ZNF667 has inhibitory effects on the induced expression and translocation of Bax protein, it should have inhibitory effects on Dox/H_2_O_2_-induced apoptosis, which was also verified by our experiments as expected. Therefore, all the evidence obtained here supports ZNF667 is a protective factor against Dox/H_2_O_2_ stress in cardiomyocytes. The molecular mechanism is involved in its inhibiting Bax expression. Although Our previous studies have implied that Mipu1 may confer cytoprotection [1], [5], , it is in this study that we for the first time shows that ZNF667/Mipu1 is a cytoprotective protein in H9c2 cardiomyocytes at least during H_2_O_2_/Dox stress.

The results discussed in this research suggest that ZNF667 is a transcription repressor of the rat Bax gene, protecting cardiomyocytes against Dox/H_2_O_2_-induced apoptosis. However, Bax and apoptosis are associated with cancer [37]-[40]. In cancer cells, an increase in Bax expression will promote apoptosis. As the last six zinc fingers of the rat ZNF667 are sufficient for its DNA binding, we compared the fingers of ZNF667 from rat with that from human, it was found that the identities are 93%, indicating that human ZNF667 may also have the same DNA binding sequence. Bio- informatic analysis shows that the human Bax promoter contains a ZNF667 DNA binding sequence, which implies that human ZNF667 may also repress human Bax expression. If this is true, ZNF667 may be involved in cancer. Interestingly, it was also found that human ZNF667 was indeed upregulated in human brain astrocytomas [41]. Of course, the roles of ZNF667 in cancer need to be deeply elucidated in the future.

Collectively, our present study demonstrated that ZNF667 is a novel direct regulator of the rat Bax gene and a protective protein in cardiomyocytes, which has the potential to protect the rat heart against ischemia/reperfusion injury. Our findings also provided new clues for ZNF667 functions in other processes or diseases .

## Supporting Information

Figure S1 **ZNF667 inhibits Dox-induced expression of the rat Bax gene.** Cells were treated with different doses of Dox for 12 h (A) or with 1 µM Dox for the indicated times (B) or transfected with the indicated vector for 24 h and treated with 1 µM Dox for 12 h (C), or co-transfected with the reporter construct pBa-luc (0.5 µg) and pRL-TK (0.02 µg) plus pcDNA3.1 (0.5 µg) or pcDNA3.1-ZNF667 (0.5µg), or pRNA-U6.1 (sC, 0.5µg) or pRNA-U6.1-ZNF667 (0.5µg), and serum starved in DMEM overnight followed by treatment with 1 µM Dox for 12 h (D). Total RNA was extracted from cells of each group, and qRT-PCR was performed to quantify Bax mRNA expression levels, shown as the relative difference from the control or pcDNA3.1 or control siRNA normalized to GAPDH expression levels (n = 3), respectively. The reporter activities are shown as the relative luciferase activity normalized to the pRL-TK activity (n = 8). *P<0.05 vs. pcDNA3.1; #P<0.05 vs. ctrl siRNA; $P<0.05 vs. pcDNA3.1+ Dox; &P<0.05 vs. ctrl siRNA + Dox. P, pcDNA3.1; Z, pcDNA3.1-ZNF667; Cs, control siRNA; Zs, ZNF667 siRNA; Dox, doxorubicin; Luc, luciferase.
(TIF)

Figure S2 **ZNF667 inhibits Bax mitochondrial translocation induced by H_2_O_2_.** Cells were transfected with pcDNA3.1 or pcDNA3.1-ZNF667 (A), and transfected with pRNA-U6.1 or pRNA-U6.1-ZNF667 (B), respectively, for 24 h. The cells were then treated with H_2_O_2_ for 6 h or untreated. The cell mitochondria was prepared using a kit. The mitochondrial proteins were subjected to Western blot analyses using the antibodies indicated. For both A and B, shown in the bottom panel are representative results, and shown in the top panel are the means ±SEM of three independent experiments. *P<0.05 vs. pcDNA3.1; #P<0.05 vs. pcDNA3.1+ H_2_O_2_; $P<0.05 vs. siRNA Ctrl; &P<0.05 vs. siRNA Ctrl + H_2_O_2_. VDAC, voltage-dependent anion channel protein.
(TIF)

Figure S3 **Target detection assay revealed the last zinc fingers of ZNF667 is required and sufficient for its DNA binding.** **A**. Scheme of ZNF667 and its truncated fusions. GST was fused to their N-termini. **B and D.** Purified GST-ZF, GST-ZF2, GST-ZF1 and GST were separated on 10% SDS–PAGE, as indicated on the top and stained with Coomassie blue. **C and E**. A target detection assay was performed in the presence of zinc after the same proteins in B and D, respectively, were transferred to two nitrocellulose membranes. After the membranes were incubated with biotin-labeled probes and washed, the bound DNA was visualized using a Lightshift chemiluminescent EMSA kit (Pierce). The target detection assay revealed zinc-dependent binding of the ZF and the ZF2 to the probe (indicated by arrows). This shows the last six zinc fingers of ZNF667 (ZF2) is sufficient and necessary for its binding to the DNA binding sites. GST, glutathione S-transferase.
(TIF)

Figure S4 **ZNF667 prevents apoptosis and caspase-3 activation mediated by Dox in H9c2 cells.** The cells transfected with the indicated plasmids were treated or untreated with Dox for 6 h. The cells were harvested by trypsinization, and then used for apoptosis analysis by flow cytometry (A) or for caspase-3 activity analysis by a kit (B). #P<0.05 vs. Ctrl siRNA; $P<0.05 vs. pcDNA3.1+ Dox; &P<0.05 vs. Ctrl siRNA + Dox. P, pcDNA3.1; Z, pcDNA3.1-ZNF667; Cs, control siRNA; Zs, ZNF667 siRNA.
(TIF)
