# Supplementary figures and images for: ZNF667/Mipu1 Is a Novel Anti-Apoptotic Factor That Directly Regulates the Expression of the Rat Bax Gene in H9c2 Cells
Source: PLoS One. 2014 Nov 14;9(11):e111653. doi: 10.1371/journal.pone.0111653 (PMC4232351; doi:10.1371/journal.pone.0111653)

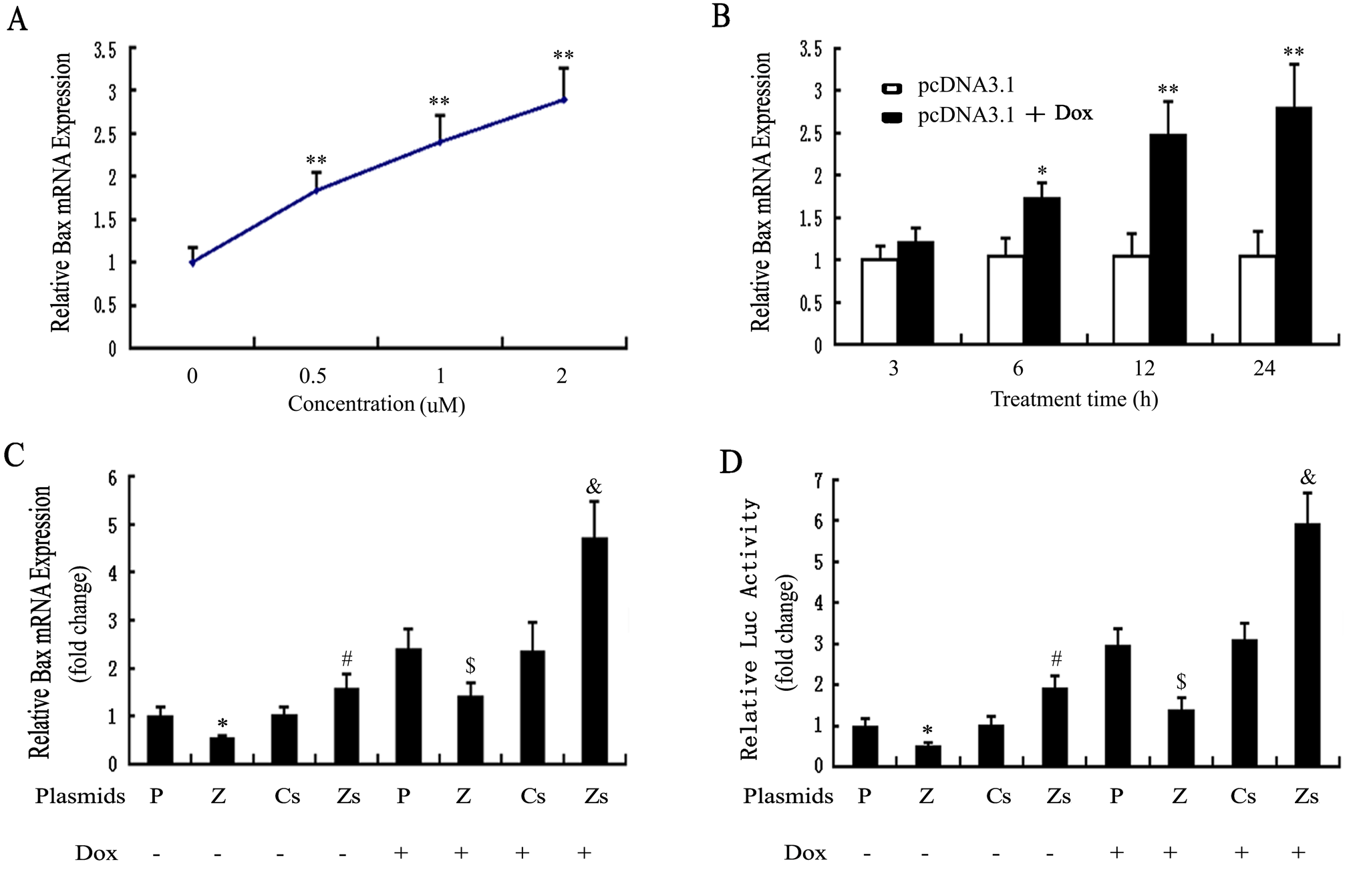

Supplement: Figure S1 — ZNF667 inhibits Dox-induced expression of the rat Bax gene. Cells were treated with different doses of Dox for 12 h (A) or with 1 µM Dox for the indicated times (B) or transfected with the indicated vector for 24 h and treated with 1 µM Dox for 12 h (C), or co-transfected with the reporter construct pBa-luc (0.5 µg) and pRL-TK (0.02 µg) plus pcDNA3.1 (0.5 µg) or pcDNA3.1-ZNF667 (0.5µg), or pRNA-U6.1 (sC, 0.5µg) or pRNA-U6.1-ZNF667 (0.5µg), and serum starved in DMEM overnight followed by treatment with 1 µM Dox for 12 h (D). Total RNA was extracted from cells of each group, and qRT-PCR was performed to quantify Bax mRNA expression levels, shown as the relative difference from the control or pcDNA3.1 or control siRNA normalized to GAPDH expression levels (n = 3), respectively. The reporter activities are shown as the relative luciferase activity normalized to the pRL-TK activity (n = 8). *P<0.05 vs. pcDNA3.1; #P<0.05 vs. ctrl siRNA; $P<0.05 vs. pcDNA3.1+ Dox; &P<0.05 vs. ctrl siRNA + Dox. P, pcDNA3.1; Z, pcDNA3.1-ZNF667; Cs, control siRNA; Zs, ZNF667 siRNA; Dox, doxorubicin; Luc, luciferase. (TIF) [file pone.0111653.s001.tif]

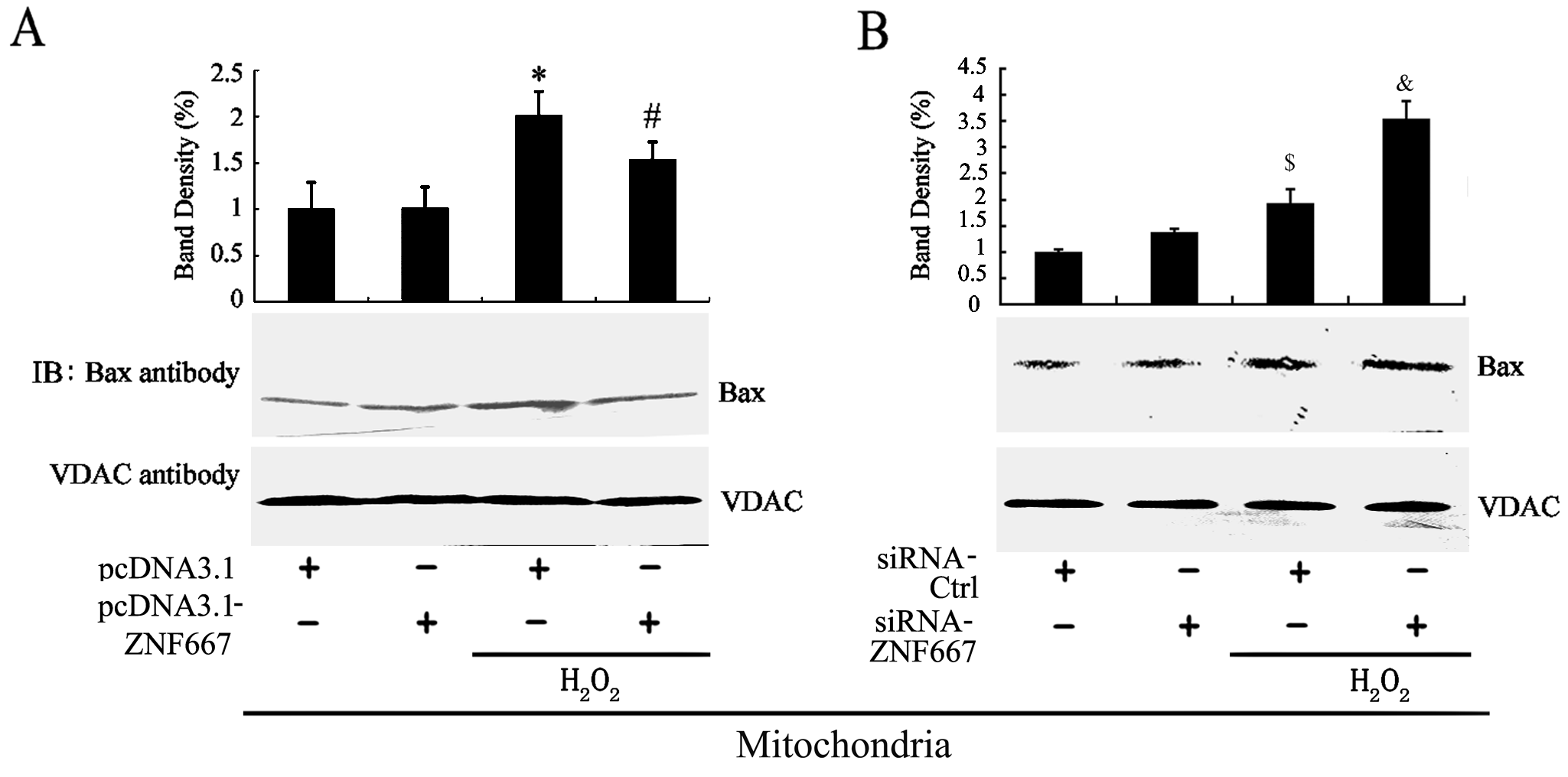

Supplement: Figure S2 — ZNF667 inhibits Bax mitochondrial translocation induced by H2O2. Cells were transfected with pcDNA3.1 or pcDNA3.1-ZNF667 (A), and transfected with pRNA-U6.1 or pRNA-U6.1-ZNF667 (B), respectively, for 24 h. The cells were then treated with H2O2 for 6 h or untreated. The cell mitochondria was prepared using a kit. The mitochondrial proteins were subjected to Western blot analyses using the antibodies indicated. For both A and B, shown in the bottom panel are representative results, and shown in the top panel are the means ±SEM of three independent experiments. *P<0.05 vs. pcDNA3.1; #P<0.05 vs. pcDNA3.1+ H2O2; $P<0.05 vs. siRNA Ctrl; &P<0.05 vs. siRNA Ctrl + H2O2. VDAC, voltage-dependent anion channel protein. (TIF) [file pone.0111653.s002.tif]

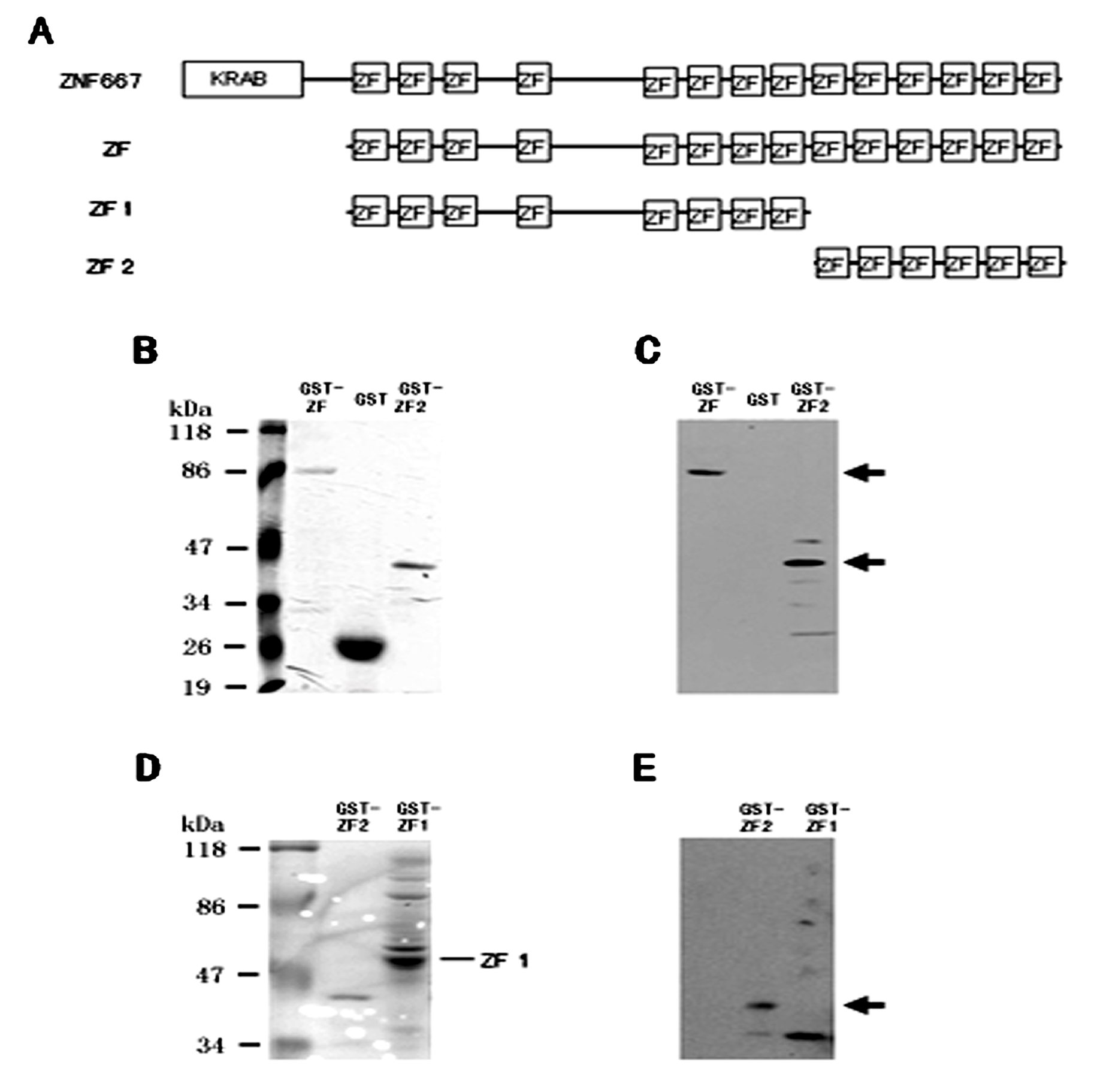

Supplement: Figure S3 — Target detection assay revealed the last zinc fingers of ZNF667 is required and sufficient for its DNA binding. A. Scheme of ZNF667 and its truncated fusions. GST was fused to their N-termini. B and D. Purified GST-ZF, GST-ZF2, GST-ZF1 and GST were separated on 10% SDS–PAGE, as indicated on the top and stained with Coomassie blue. C and E. A target detection assay was performed in the presence of zinc after the same proteins in B and D, respectively, were transferred to two nitrocellulose membranes. After the membranes were incubated with biotin-labeled probes and washed, the bound DNA was visualized using a Lightshift chemiluminescent EMSA kit (Pierce). The target detection assay revealed zinc-dependent binding of the ZF and the ZF2 to the probe (indicated by arrows). This shows the last six zinc fingers of ZNF667 (ZF2) is sufficient and necessary for its binding to the DNA binding sites. GST, glutathione S-transferase. (TIF) [file pone.0111653.s003.tif]

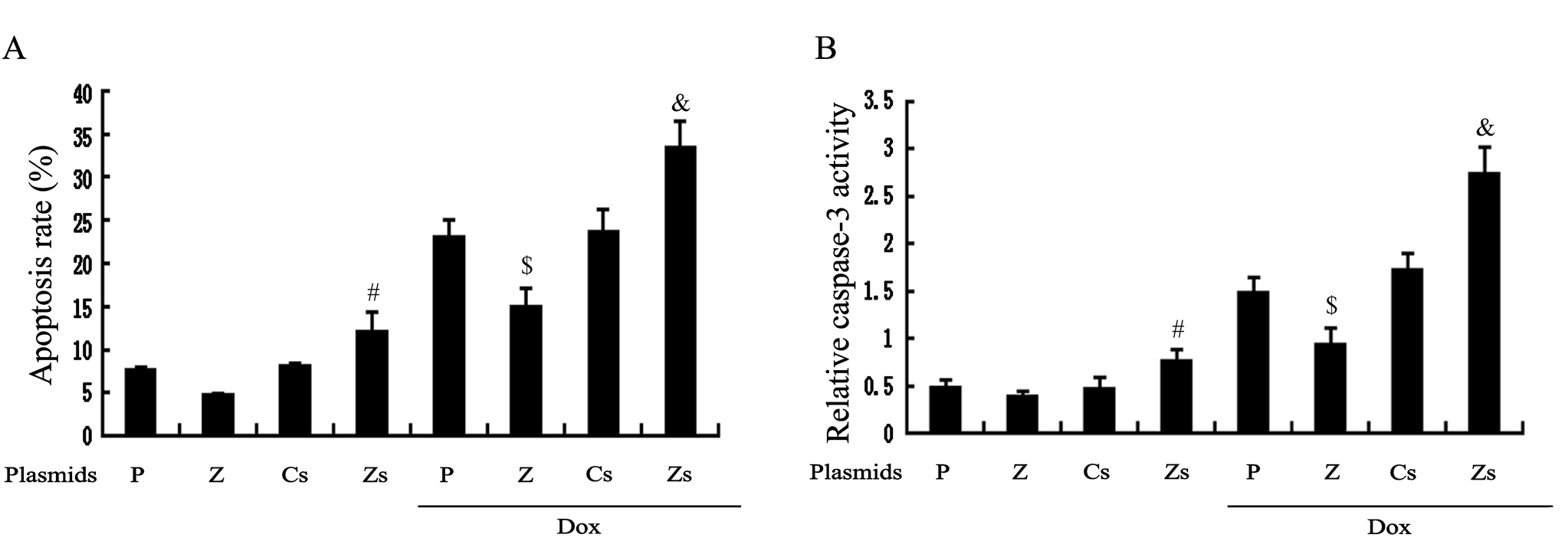

Supplement: Figure S4 — ZNF667 prevents apoptosis and caspase-3 activation mediated by Dox in H9c2 cells. The cells transfected with the indicated plasmids were treated or untreated with Dox for 6 h. The cells were harvested by trypsinization, and then used for apoptosis analysis by flow cytometry (A) or for caspase-3 activity analysis by a kit (B). #P<0.05 vs. Ctrl siRNA; $P<0.05 vs. pcDNA3.1+ Dox; &P<0.05 vs. Ctrl siRNA + Dox. P, pcDNA3.1; Z, pcDNA3.1-ZNF667; Cs, control siRNA; Zs, ZNF667 siRNA. (TIF) [file pone.0111653.s004.tif]
